# Ectoparasite-borne *Bartonella* and *Rickettsia* in Chilean populations of *Rattus rattus*: prevalence, genetic diversity and environmental associations

**DOI:** 10.1017/S0031182025101406

**Published:** 2026-02

**Authors:** Elaine Monalize Serafim de Castro, Ananda Müller, Ricardo Gutiérrez, María Carolina Silva-de la Fuente, Sebastián Muñoz-Leal, Mario Espinoza-Carniglia, Lucila Moreno

**Affiliations:** 1 Departamento de Ciencia Animal, Facultad de Ciencias Veterinarias, Universidad de Concepción, Chillán, Chile; 2 Biomedical Sciences, Lewyt College of Veterinary Medicine, Long Island University, 720 Northern Boulevard, Greenvale, NY 11546, USA; 3 Biomedical Sciences Department, One Health Center for Zoonoses and Tropical Veterinary Medicine, Ross University, School of Veterinary Medicine, West Farm, Saint Kitts and Nevis; 4 Departamento de Ciencias Agrarias, Facultad de Ciencias Agrarias y Forestales, Universidad Católica del Maule, Curicó, Chile; 5 Centro de Estudios Parasitológicos y de Vectores, Universidad Nacional de La Plata, La Plata, Argentina; 6 Departamento de Zoología, Facultad de Ciencias Naturales y Oceanográficas, Universidad de Concepción, Concepción, Chile

**Keywords:** *Bartonella*, ectoparasites, *Rickettsia*, synanthropic rodents

## Abstract

*Rattus rattus* is a known reservoir of zoonotic pathogens, including *Bartonella* and *Rickettsia*, transmitted by ectoparasites such as fleas, mites, lice and ticks. The circulation of *Bartonella* and *Rickettsia* in these vectors in Chile remains poorly characterized. To evaluate the association between ectoparasite abundance, prevalence and diversity (including lice, fleas, mites and ticks) and the presence of *Bartonella* and *Rickettsia* within ectoparasites collected from *R. rattus* across different anthropogenic gradients in Chile, a total of 1,339 ectoparasites were collected from 411 *R. rattus* individuals across 27 localities. Ectoparasites were identified morphologically, and molecular detection of bacteria was performed using conventional and qPCR, targeting multiple genetic markers. Haplotype diversity and phylogenetic relationships were assessed. *Bartonella* and *Rickettsia* DNA were detected in fleas, ticks, mites and lice of *R. rattus*, with prevalence values reported separately for pooled and individually analysed ectoparasites. *Bartonella tribocorum, B. rochalimae* and *B. mastomydis* were identified. *Rickettsia felis* was confirmed in multiple ectoparasite groups. High haplotype diversity was observed in *Bartonella* but not in *Rickettsia*. Urbanization and tick prevalence were negatively associated with *Bartonella* occurrence; flea and tick prevalences were negatively associated with *Rickettsia. Rattus rattus* and their ectoparasites harbour a diverse range of potentially zoonotic *Bartonella* and *Rickettsia* species. These findings highlight the need for integrated surveillance and vector control strategies, especially in areas with variable human-wildlife interaction.

## Graphical abstract

**Figure:**
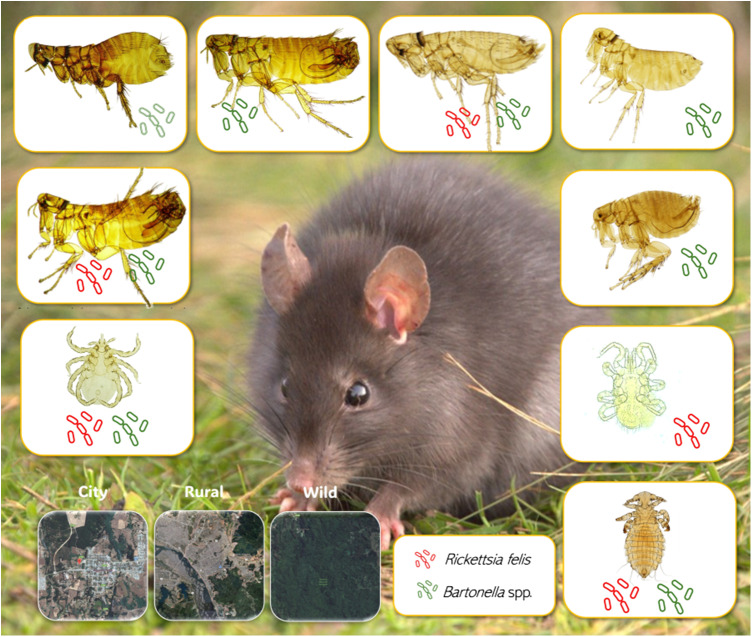

## Introduction

Synanthropic rodents cohabiting with humans, including *Rattus rattus, Rattus norvegicus* and *Mus musculus*, are of great public health importance (Steppan et al., 2005; Himsworth et al., 2013), as they can carry at least 66 zoonotic diseases transmitted by viruses, bacteria, fungi, helminths and protozoa (Han et al., 2015). Fleas, mites and lice are the most prevalent ectoparasites in rats and mice, while ticks, although important vectors of infectious agents, do not usually parasitize synanthropic rodents (Berdoy et al., 1995; Mihalca et al., 2012; Hornok et al., 2015; Chakma et al., 2017). Among the bacteria with pathogenic potential transmitted by vector-associated arthropods, those of the genus *Bartonella* and *Rickettsia* stand out (Eisen and Gage, 2012). However, despite the wide distribution of rodents and their importance as vectors in public health, there are still gaps in knowledge about the role of ectoparasites in transmitting these bacteria.

According to Armién et al. (2016), the presence of these bacteria is related to environments with anthropogenic disturbance. Urban expansion modifies the structure of rodent communities, favouring generalist and synanthropic species to the detriment of specialized native wild rodents (Faeth et al., (2005); Bradley and Altizer, 2007). Encroachment into wilderness areas due to increasing human population expansion leads to a greater likelihood of contact with infected ectoparasites (Moreno-Salas et al., 2019; Fantozzi et al., 2021) increasing the probability of disease transmission (Dickman and Doncaster, 1987; Hassell et al., 2017).

Conversely, rural areas play a crucial role in the epidemiology of infectious diseases, acting as points of interaction between wild and domestic species, including humans (López Berrizbeitia et al., 2024). This generates a higher probability of disease emergence, particularly bacterial, which can then spread to both urban and wild environments (Neiderud, 2015).

*Bartonella* spp. are classified as intracellular Gram-negative bacteria, mainly transmitted by rodents, which serve as important reservoirs (Ying et al., 2002; Favacho Arm de et al., 2015; Gonçalves et al., 2016). Ectoparasites, like fleas, mites and lice, may act as vectors (Colborn et al., 2010; Klangthong et al., 2015; Moreno-Salas et al., 2019). These bacteria are responsible for numerous emerging or re-emerging infectious diseases, with clinical manifestations that vary depending on the type of infection and the immune status of the patient (Tahmasebi Ashtiani et al., 2025). They primarily infect erythrocytes and epithelial cells, and exhibit mechanisms for evading the host immune system (Harms and Dehio, 2012).

Another zoonotic disease transmitted by rodents involves bacteria of the genus *Rickettsia*, which are transmitted by fleas that infest rodents, birds, cats and occasionally humans (Renvoisé et al., 2009). Rickettsiae are obligate intracellular Gram-negative bacteria that cause rickettsioses such as Spotted Fever and Murine Typhus (Gillespie et al., 2008).

Given that 75% of human diseases are of zoonotic origin (Taylor et al., 2001), understanding the epidemiology of rodent ectoparasites is crucial. Although there is extensive literature on synanthropic rodents worldwide, data on the pathogens carried by ectoparasites in South America remain scarce (González-Acuña and Guglielmone, 2005; Lareschi and Krasnov, 2010; Nava and Lareschi, 2012; López Berrizbeitia et al., 2015; Lareschi et al., 2016; Nava et al., 2017; Moreno-Salas et al., 2019).

This study aims to evaluate the association between ectoparasite abundance, prevalence and diversity – including lice, fleas, mites and ticks – and the presence of *Bartonella* and *Rickettsia* in ectoparasites collected from *R. rattus* across varying anthropogenic gradients in Chile. It is hypothesized that ectoparasite abundance, prevalence and diversity are positively associated with the presence of *Bartonella* and *Rickettsia* within these ectoparasites, and that these associations differ according to the level of anthropogenic disturbance across localities in Chile.

## Materials and methods

### Areas of study

**Figure 1.**
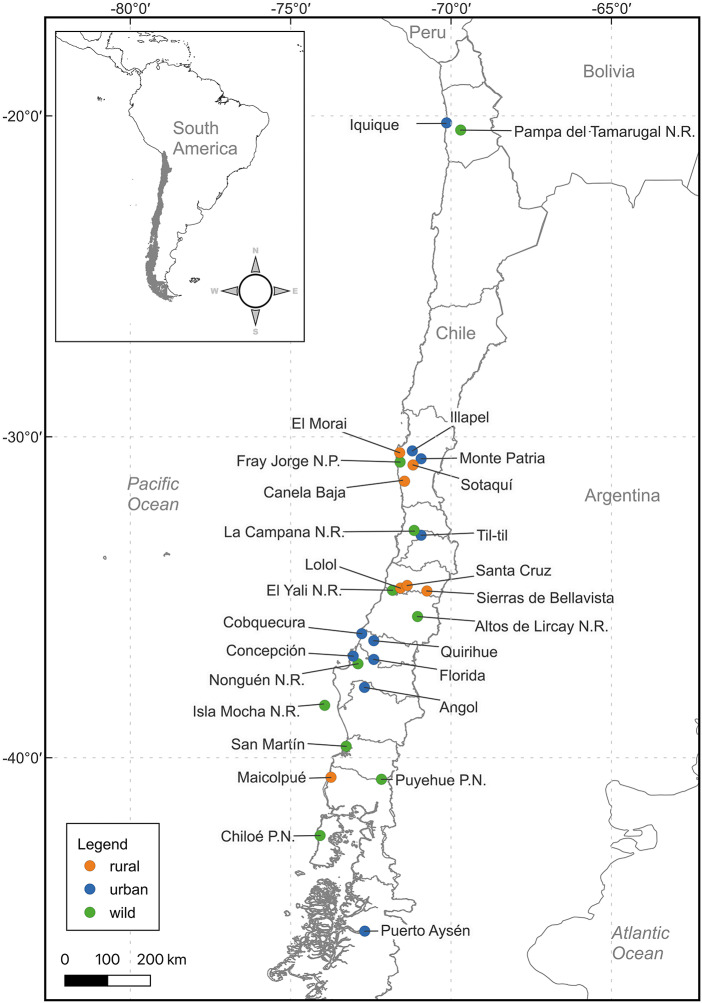
Map of Chile indicating the rodent sampling locations, subdivided; blue: urban zone (city), green: rural zone, orange: wild zone.

A total of 27 localities in Chile were analysed (Figure 1), classified according to their degree of anthropization, following the definitions of the National Institute of Statistics: ‘City’ (urban entity with more than 5,000 inhabitants) and ‘Rural’ (rural entity with a population between 2,001 and 5,000 inhabitants, or between 1,001 and 2,000 inhabitants, which meets the economic activity criteria of INE, 2005). The natural areas (‘Wild’) evaluated consisted of Parks and Reserves administered by CONAF (Corporación Nacional Forestal, Chile).

Live rodents were captured using Sherman traps baited with oats over 2 consecutive nights at each sampling site in 4 seasons (summer, autumn, winter, spring). To obtain a larger coverage area, transects equipped with 200 traps were set up, spaced 10 m apart, resulting in a total sampling effort of 4,800 traps per night. This allowed for a more representative sampling of the rodent population in each locality studied, considering seasonal variations.

**Figure 2.**
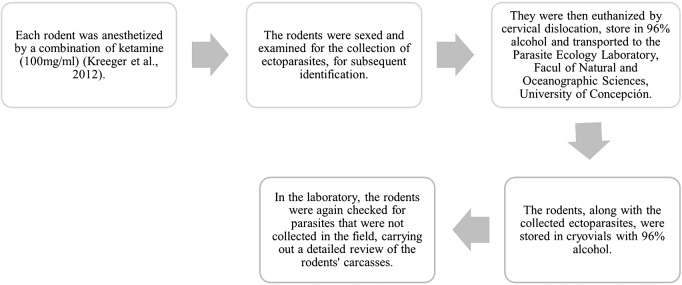
Methodology used to ensure the welfare of the animals.

Each captured rodent was identified using taxonomic guides (Iriarte, 2008). Figure 2 illustrates the methodology used to ensure animal welfare and facilitate handling. All handling and euthanasia of the animals followed protocols for field and laboratory studies with rodents (Herbreteau et al., 2011), approved by the Ethics Committee of the Vice-Rectory of Research and Development of the Universidad de Concepción. Rodent captures were authorized by the Agricultural Service (SAG R.E: 8968-2015, 1657-2016, 73-2016, 23-2017) and the National Forestry Corporation (CONAF Nº 018-2015).

### Mounting and identification of ectoparasites

Fleas collected were prepared following the modified methodology of Hastriter and Whiting (2009). Mites and ticks (nymph and larvae) were rinsed in Nesbitt’s solution (40 g chloral hydrate, 25 mL distilled water and 2.5 mL concentrated HCL) for 72 h and then mounted as permanent preparations using Berlese’s solution (Krantz and Walter, 2009). Lice were treated with 20% KOH for initial deposition, stained with alcohol-phenol-eosin, clarified using alcohol-phenol, and permanently mounted using Canada balsam (Price et al., 2003).

Ectoparasites were identified using taxonomic characteristics and specific descriptions for each group. Identification references included Hopkins and Rothschild (1962), Hopkins (1956), Hopkins and Rothschild (1966), Johnson (1957), Sánchez and Lareschi (2014) and López Berrizbeitia et al. (2015) for fleas, Brennan and Goff (1977), Furman (1972) and Radovsky (2010) for mites, Castro (1982), Castro and González (1996) and Gómez (1998) for lice, and Keirans and Clifford (1978), Guglielmone et al. (2004), Barros-Battesti et al. (2013), and Nava et al. (2017) for ticks.

### Estimation of parasitological descriptors and ectoparasite diversity

Mean abundance (MA), mean intensity (MI) and prevalence (P%) were calculated using Quantitative Parasitology 3.0 software. The 95% confidence intervals were determined using the bootstrap method (2,000 replicates). The bootstrap *t*-test was applied to compare MA and MI between seasons and anthropization zones, while Fisher’s exact *t*-test was used to analyse P% data. Due to the low number of samples and the lack of significant differences between seasons regarding ectoparasites presence, seasons were grouped.

Diversity was estimated using the Shannon–Wiener index (H’), which considers both species richness and relative abundance in the host community. Only species with more than 10 individuals were included for statistical analysis.

### Molecular identification of *Bartonella* and *Rickettsia spp*. in ectoparasites

Collected ectoparasites underwent DNA extraction individually (adult ticks and fleas) or in pools of up to 10 individuals per species (tick nymphs, mites and lice). Since it was necessary to identify ectoparasites morphologically prior to DNA extraction, different treatments were applied to the different groups of ectoparasites. For fleas, specimens were cut in half between the third and fourth abdominal tergites using a sterilized scalpel, taking care to change the scalpel between each sample. After DNA extraction, the exoskeleton was recovered and mounted following the previously described protocols. For adult ticks, individuals were identified under a stereomicroscope before proceeding with DNA extraction after maceration. In the case of nymphs and larvae, where extraction was performed in pools for each group, individuals were first examined under a stereomicroscope; some were mounted and identified, while the remaining specimens were grouped in pools and macerated for DNA extraction. For lice, a similar procedure was followed: specimens were examined under a stereomicroscope, selected individuals were mounted and identified, and the remaining ones were subjected to DNA extraction. The parasite pools were determined by host individual.

DNA was extracted using the DNeasy Blood & Tissue® kit (Qiagen, Hilden, Germany) following the manufacturer’s instructions. DNA concentrations were quantified using a Nanodrop TM 2000 spectrophotometer (Thermo Fisher TM), obtaining values between 50 and 200 ng/μL.

To determine the presence of *Bartonella* and *Rickettsia* species, real-time PCR (qPCR) screening with SYBR Green was performed. Detailed molecular characterization was subsequently conducted using conventional PCR (cPCR) followed by sequencing of the amplified products. Endogenous gene amplification for all ectoparasites DNA was performed via cPCR for detection of eukaryotic 18S rRNA DNA using the following primer sequences: F-573: CGC GGT AAT TCC AGC TCC A and R-1200: CCC GTG TTG AGT CAA ATT AAG C (Hadziavdic et al., 2014).

For *Bartonella* spp. and *Rickettsia* spp. detection, ectoparasite samples were processed in duplicate for qPCR. Cycles were performed on the CFX96 Touch™ Real-Time PCR Detection System CFX96 thermal cycler (BioRad, Hercules, CA, USA) with Low-Profile Multiplate™ PCR plates (BioRad©, Hercules, CA). For the detection of *Bartonella* spp. qPCR first targeted the *nuoG* fragment (Colborn et al., 2010). Positive samples were submitted to conventional PCR amplification targeting the *gltA* (citrate synthase), *rpoB* (subunit RNA polymerase) genes and *ITS* intergenic region (16S-23S rRNA) (Pitulle et al., 2002; Maggi and Breitschwerdt, 2005; Paziewska et al., 2011; Moreno-Salas et al., 2019; Müller et al., 2020). Details of primers and amplified fragment size for each gene are provided in Table 1.

**Table 1.** Targets genes and primers sets used for conventional PCR for detections of *Bartonella* spp. and *Rickettsia* spp. in this study

Target (Amplicon size)	Primer (Forward or Reverse)	Nucleotide Sequence (5´–3´)	Reference
*Bartonella* spp.			
*gltA* (767)	CS443f (F)	GATCYTCAATCATTCTTTCCA	Pitulle et al., 2002
	Cs1210r (R)	GATCYTCAATCATTTCTTTCCA	
*rpoB* (333)	rpoBF (F)	GCACGATTYGCATCATCATTTTCC	Paziewska et al., 2011
	rpoBR (R)	CGCATTATGGTCGTATTTGTCC	
ITS 453-717	325s (F)	CTTCAGATGATGATCCCAAGCCTTYTGGCG	Diniz et al., 2007
	1100as (R)	GAACCGACGACCCCCTGCTTGCAAAGCA	
*Rickettsia* spp.			
*gltA* (401)	CS-78 (F)	GCAAGTATCGGTGAGGATGTAAT	Labruna et al., 2004
	CS-323 (R)	GCTTCCTTAAAATTCAATAAATCAGGAT	
*ompA* (512)	Rr190.70p (F)	ATGGCGAATATTTCTCCAAAA	Regnery et al., 1991
	Rr190.602 n (R)	AGTGCAGCATTCGCTCCCCCT	
*ompB* (475-267)	1Rc.rompB.4362p (F)	GTCAGCGTTACTTCTTCGATGC	Choi et al., 2005
	2Rc.rompB.4836 n (R)	CCGTACTCCATCTTAGCATCAG	
	3Rc.rompB.4496p (F)	CCAATGGCAGGACTTAGCTACT	
	4Rc.rompB.4762 n (R)	AGGCTGGCTGATACACGGAGTAA	

For the detection of *Rickettsia* spp. qPCR amplified a fragment of citrate synthase (*gltA*) gene following the protocol of Stenos et al. (2005), with some modifications. The qPCR amplified *gltA* using cycles at 95 °C for 1 min, followed by 40 cycles at 95 °C for 15 sec and 60 °C for 1 min. PCR was then conducted for positive samples targeting the *gltA, ompA* and *ompB* (outer membrane protein A and B) genes (Table 1).

To calculate the frequency of bacterial DNA detection in *Bartonella* and *Rickettsia* within each ectoparasite group and locality, a sample was considered positive if at least 1 of 3 specific genes was amplified (*gltA, ITS* and *rpoB* for *Bartonella; gltA, ompA* and *ompB* for *Rickettsia*).

Sequencing of conventional PCR amplicons was performed by MACROGEN (Seoul, Korea). Sequences were aligned and assembled into a contig, and primer sequences were trimmed using MEGA v.7 software (Kumar et al., 2016). Sequence alignments were performed using MUSCLE (Edgar, 2004). Ambiguities were resolved manually by analysing chromatograms. Clean sequences were initially analysed by BLASTn using the MegaBLAST algorithm in NCBI (National Center for Biotechnology Information) to confirm species identity and place our sequences within the broader genetic context of *Bartonella* and *Rickettsia* (Morgulis et al., 2008; Chen et al., 2015).

A maximum likelihood phylogenetic tree was constructed for each gene (*gltA, ITS* and *rpoB* for *Bartonella; ompB* for *Rickettsia*), using IQ-TREE2 (Minh et al., 2020), and *Brucella abortus* was used as an outgroup for *Bartonella* trees, except for the *ITS* gene, for which *Bartonella bacilliformis* was used. The *Rickettsia* tree was rooted with *Rickettsia bellii*. A best-fit model and bootstrap support based on 1,000 replicates were applied.

Genetic diversity within *Bartonella* and *Rickettsia* populations was assessed using specialized tools. Haplotype identification was performed using DnaSP v6.11.01 (Rozas et al., 2017). Additionally, we implemented a methodology for constructing haplotype networks using R software (Paradis, 2010; R Core Team, 2022). Haplotypes were named sequentially (H1, H2, etc.) according to their frequency in the dataset. Names do not correspond to preexisting GenBank haplotypes but were assigned for the purpose of this study.

The sequences were deposited in GenBank as follows: for *Bartonella*, accession numbers PP151229–PP151235 (*gltA* sequences), PP151219–PP151228 (*rpoB* sequences), and PP150442–PP150444 (*ITS* sequences). For *Rickettsia*, accession numbers PP151242 (*gltA* sequence) and PP151236–PP151241 (*ompA* sequences).

### Association between *Bartonella* and *Rickettsia* presence with parasitological descriptors and degrees of anthropization

To assess the relationship between bacterial occurrence with degrees of anthropization (locality type), abundance, diversity and prevalence of ectoparasites, generalized linear models (GLM) were used. The presence or absence of the bacterial DNA in the ectoparasites was considered the dependent variable in a binomial distribution and logit function, while the explanatory variables included locality type (city, rural and wild), abundance and prevalence of each ectoparasite group (fleas, mites, lice and ticks), and diversity of all ectoparasites. The Chi-square test was used to evaluate the differences in the prevalence of *Bartonella* and *Rickettsia*. Values of *p* ≤ 0.05 were considered significant. These analyses were performed using the JMP software® (SAS Institute Inc., USA).

## Results

### Ectoparasites and degree of anthropization

A total of 411 *R. rattus* individuals were captured across different seasons (summer: *n* = 188; autumn: *n* = 27; winter: *n* = 168; spring: *n* = 28) and localities (city: *n* = 190; rural: *n* = 64; wild: *n* = 157). From these individuals, 1,339 ectoparasites were collected, including lice (52.48%; *n* = 697), fleas (17.6%; *n* = 236), mites (16.8%, *n* = 225) and ticks (13.5%; *n* = 181) (Table 2). Taxonomic identification yielded 2 louse species, 17 flea species, 3 mite taxa (2 identified to genus level and one to family level), and 2 tick species (Table 3).

**Table 2.** Ectoparasites collected from *Rattus rattus* according to the degree of anthropization. Prevalence (P%), mean abundance (MA) and mean intensity (MI) are given with their respective confidence intervals (CI 95)

Degree of anthropization (*n*)	Descriptors	Lice	Fleas	Mites	Ticks
City (10)	No. of rodents parasitized	41	42	23	23
	No. of ectoparasites collected	163	95	87	53
	P% (CI 95)	21.6 (0.17–0.9)	22.6 (0.16–0.28)	12.1 (0.08–0.18)	12.1 (0.08–0.18)
	MA (CI 95)	0.86 (0.54–1.32)	0.50 (0.29–0.61)	0.46 (0.25–0.90)	0.28 (0.17–0.52)
	MI (CI 95)	3.98 (2.73–5.49)	2.21 (1.60–2.55)	3.78 (2.43–6.83)	2.30 (1.65–3.91)
Rural (7)	No. of rodents parasitized	11	17	8	11
	No. of ectoparasites collected	96	61	11	38
	P% (CI 95)	17.2 (0.09–0.29)	26.6 (0.05–0.15)	12.5 (0.06–0.24)	17.2 (0.10–0.30)
	MA (CI 95)	1.50 (0.58–3.87)	0.95 (0.17–0.64)	0.17 (0.06–0.40)	0.59 (0.24–1.42)
	MI (CI 95)	8.73 (4.27–18.45)	3.59 (2.40–5.73)	1.38 (1–1.75)	3.45 (1.82–7.45)
Wild (10)	No. of rodents parasitized	68	30	32	42
	No. of ectoparasites collected	438	69	127	90
	P% (CI 95)	43.3 (0.36–0.51)	19.1 (0.14–0.26)	20.4 (0.14–0.27)	26.8 (0.03–0.05)
	MA (CI 95)	2.79 (2.03–3.91)	0.44 (0.28–0.64)	0.81 (0.48–1.39)	0.57 (0.05–0.11)
	MI (CI 95)	6.44 (4.97–8.49)	2.30 (1.73–2.93)	3.97 (2.69–6.31)	2.14 (1.71–2.81)

**Table 3.** Parasitological indices for the different areas of anthropization by species and ectoparasite group. The confidence interval (95%) is indicated in brackets to the right of each value. P%: Prevalence, MA: Mean abundance, MI: Mean intensity, C: City, R: Rural, W: Wild, *N*: number of ectoparasites

Ectoparasite	Degree of anthropization	*N*	*P* (%)	IC (95%)	MA	IC (95%)	IM	IC (95%)
Mites								
*Ornithonyssus* sp.	C	62	10	5–13	0.32	0.20–0.60	3.6	1.8–6.5
	R	8	8	3–18	0.13	0.03–0.30	1.6	1.0–2.8
	W	102	17	12–24	0.65	0.40–1.30	3.8	2.1–6.0
*Laelaps* sp.	C	17	4	2–8	0.09	0.03–0.20	2.1	1.5–3.0
	R	3	5	4–20	0.13	0–0.11	1.3	1.0–1.7
	W	13	4	4–12	0.08	0.01–0.10	1.2	1–1.45
Trombidiformes	C	8	2	0–5	0.04	0.01–0.20	2.7	1–6
	R	0	0	–	0	–	0	–
	W	12	4	2–10	0.07	2–12	1.4	1–1.9
Ticks								
*Ixodes sigelos*	C	53	12	8–17	0.31	0.18–0.54	2.6	1.8–4.2
	R	38	17	10–30	0.59	0.22–1.5	3.5	1.7–6.7
	W	87	24	40–50	0.52	0.35–0.75	6.4	5–8.6
*Ornithodoros* sp.	C	0	–	–	0	–	–	–
	R	0	–	–	0	–	–	–
	W	3	1	0–5	0.02	0–0.06	1.5	1–1.5
Lice								
*Polyplax spinulosa*	C	153	22	15–27	0.82	0.5–1.25	3.8	2.7–5.5
	R	96	17	12–32	1.5	0.5–4.1	8.73	4.3–18.8
	W	438	43	35–51	2.8	2–3.9	6.44	4.8–8.4
*Hoplopleura pacifica*	C	1	0.5	0–3	0.01	0–0.02	1	0
	R	0	0	–	0	–	0	–
	W	0	0	–	0	–	0	–
Fleas								
*Ctenoparia inopinata*	C	1	0.5	0–3	0.01	0–0.02	1	0
	R	0	0	–	0	–	0	–
	W	0	0	–	0	–	0	–
*Ctenoparia jordani*	C	0	0	–	0	–	0	–
	R	0	0	–	0	–	0	–
	W	1	0.6	0–4	0.01	0–0.02	1	0
*Delosticus coxalis*	C	0	0	–	0	–	0	0
	R	0	0	–	0	–	0	–
	W	6	1.30	0–5	0.04	0–0.10	3	0
*Delosticus smiti*	C	1	0.5	0–3	0.01	0–0.02	1	0
	R	0	0	–	0	–	0	–
	W	0	0	–	0	–	0	–
*Echidnophaga gallinacea*	C	0	0	–	0	–	0	–
	R	5	1.60	0–8	0.08	0–0.24	1	0
	W	0	0	–	0	–	0	–
*Hectopsylla* sp.	C	0	0	–	0	–	0	–
	R	0	0	–	0	–	0	–
	W	1	0.60	0–4	0.01	0–0.02	1	0
*Leptopsylla segnis*	C	25	5	3–9	0.1	0.04–0.2	1.9	1.2–2.6
	R	34	19	10–30	0.53	0.25–1	2.83	1.8–4.8
	W	2	1	0–40	0.01	0.01–0.08	1	0
*Neotyphloceras chilensis*	C	0	0	–	0	–	0	–
	R	3	3.2	0.4–11	0.03	0–0.08	1.5	1–1.5
	W	0	0	–	0	–	0	–
*Neotyphloceras pardinasi*	C	8	3	1.2–6.7	0.04	0.01–0.11	1.5	1–2.2
	R	9	0.13	3.6–19.6	0.13	0.5–0.23	1.5	1–2.2
	W	8	2.50	0.7–6.4	0.05	0.01–0.12	2.3	1.5–3
*Nosopsyllus fasciatus*	C	49	13	8–18	0.24	0.15–0.4	1.9	1.5–2.4
	R	13	11	5–20	0.2	0.08–0.45	1.9	1.14–3.14
	W	3	1	0.4–5.5	0.02–	0–0.08	1.5	1–1.5
*Plocopsylla* sp.	C	3	2	0–5	0.02	0–0.04	1	0
	R	0	0	–	0	–	0	–
	W	2	1	0–5	0.01	0–0.03	1	0
*Plocopsylla wolffsohni*	C	1	0.50	0–3	0.01	0–0.02	1	0
	R	0	0		0	–	0	–
	W	0	0		0	–	0	–
*Sphinctopsylla ares*	C	2	1	0–4	0.01	0–0.03	1	0
	R	6	5	1–13	0.09	0–0.19	2	0
	W	36	9	5–15	0.23	0.1–0.4	2.6	1.6–3.5
*Pullex irritans*	C	0	0	–	0	–	0	–
	R	0	0	–	0	–	0	–
	W	4	2	0–6	0.03	0–0.06	1.3	1–1.7
*Tetrapsyllus rhombus*	C	0	0	–	0	–	0	–
	R	0	0	–	0	–	0	–
	W	2	1	0–5	0.01	0–0.03	1	0
*Tetrapsyllus* sp.	C	0	0	–	0	–	0	–
	R	0	0	–	0	–	0	–
	W	6	3	2–70	0.04	0.01–0.08	1.2	1–1.4
*Xenopsylla cheopis*	C	5	5	0–3	0.03	0–0.08	5	0
	R	0	0	–	0	–	0	–
	W	0	0	–	0	–	0	–

Regarding lice, the 2 species identified, *Polyplax spinulosa* and *Hoplopleura pacifica*, correspond to native *Rattus* species. *Polyplax spinulosa* was the dominant species, detected in 15 of the 27 sampled localities, whereas *H. pacifica* was found in only one. *Polyplax spinulosa* showed higher prevalence in wild localities compared with rural and city areas (*p* = 0.001), with no difference between rural and cities (*p* = 0.48). Mean abundances and intensity of *P. spinulosa* were also significantly greater in wild than cities (MA: *p* = 0.001; MI: *p* = 0.002) but did not differ significantly between wild and rural (MA: *p* = 0.1; MI: *p* = 0.65) or between rural and cities (MA: *p* = 0.37; MI: *p* = 0.26).

Three species commonly associated with *Rattus* hosts were identified: *Leptopsylla segnis, Nosospsyllus fasciatus* and *Xenopsylla cheopis. Leptopsylla segnis* and *N. fasciatus* were widely distributed, occurring in 8 and 11 of the 27 localities, respectively. Whereas *X. cheopis* was found only in a single city in the extreme north of Chile (Iquique). *Leptopsylla segnis* was significantly more prevalent in rural localities than in wild or city sites (*p* = 0.02), although no differences in mean abundance or intensity were detected across locality types (P%: *p* = 0.6; MA: *p* = 0.1; MI: *p* = 0.1) (Table 3). In contrast, *N. fasciatus* showed higher prevalence and mean abundance in the cities than in wild areas (P%: *p* = 0.001; MA: *p* = 0.005), and its prevalence was also greater in wild than rural localities (P%: *p* = 0.003). However, no significant differences in mean intensity were observed between cities and wild areas (MI: *p* = 0.2) nor in any parasitological descriptors significantly different between cities and rural localities (P%: *p* = 0.8; MA: *p* = 0.7; MI: *p* = 0.9). Likewise, rural and wild localities did not differ significantly in abundance or intensity for this species (MA: *p* = 0.15; MI: *p* = 0.67) (Table 3).

For mites, *Ornithonyssus* sp. was de most abundant and prevalent species. However, no significant differences were detected among locality anthropization types, whether comparing wild and rural (AM: *p* = 0.07; P%: *p* = 0.13; IM: *p* = 0.12), wild and cities (AM: *p* = 0.2; P%: *p* = 0.07; IM: *p* = 0.74) and cities and rural (AM: *p* = 0.1; P%: *p* = 0.8; IM: *p* = 0.08) (Table 3).

Regarding ticks, only *Ixodes sigelos* (*n* = 173) and larvae of *Ornithodoros* sp. (*n* = 3) were identified. No significant differences were observed in the prevalence, mean abundance and mean intensity of *I. sigelos* between wild and rural localities (P%: *p* = 0.2; MA: *p* = 0.9; MI: *p* = 0.4) or between wild and cities (P%: *p* = 0.3; MA: *p* = 0.4; MI: *p* = 0.5) and rural and cities localities (P%: *p* = 0.3; MA: *p* = 0.4; MI: *p* = 0.5) (Table 3).

### Detection and prevalence of *Bartonella* and *Rickettsia*

*Bartonella* and *Rickettsia* DNA were detected in all ectoparasite groups. Prevalence estimates are reported as pool prevalence for lice, mites and immature ticks (processed in pools of 10 specimens per host), and as individual prevalence for adult fleas and ticks (Table 4). In lice were tested in 99 pools, of which 33.3% (33/99; 95% CI: 20–45%) were positive for *Bartonella* and 19.2% (19/99; 95% CI: 9–29%) for *Rickettsia*. In mites, 22.0% (12/54; 95% CI: 8–36%) were positive for *Bartonella*, and 23.2% (23/54; 95% CI: 13–33%) for *Rickettsia.* Among ticks (*n* = 90; adults analysed individually, larvae and nymphs in pools), *Bartonella* DNA was detected in 26.6% (24/90; 95% CI: 14–38%) and *Rickettsia* DNA in 24.4% (22/90; 95% CI: 13–33%). In fleas, which were analysed individually (*n* = 193), *Bartonella* DNA prevalence was 53.4% (103/193; 95% CI: 44–63%) and *Rickettsia* DNA 50.3% (97/193; 95% CI: 49–50%).

**Table 4.** Prevalence of *Bartonella* and *Rickettsia* DNA in ectoparasite groups collected from localities with different degrees of anthropization. Detection was considered positive when at least one of the 3 target genes was amplified via qPCR (*Bartonella*: *gltA*, *ITS*, *rpoB*; *Rickettsia*: *gltA*, *ompA*, *ompB*). Prevalence is expressed as percentage and number of positive samples (in parenthesis)

Degree of Anthropization	Ectoparasite Group	Samples analyzed (pools [P]/Individuals [I])	*Bartonella* % (*n*)	*Rickettsia* % (*n*)
City	Lice	34 (P)	14.7 (5)	5.9 (2)
	Fleas	81(I)	49.3 (40)	50.6 (41)
	Mites	23 (P)	13 (3)	43.5 (10)
	Ticks	31 (P/I)	19.3 (6)	19.3 (6)
Rural	Lice	9 (P)	55.5 (5)	22.2 (2)
	Fleas	59 (I)	55.9 (33)	61 (36)
	Mites	9 (P)	33.3 (3)	66.7 (6)
	Ticks	12 (P/I)	25 (3)	16.7 (2)
Wild	Lice	56 (P)	30.4 (17)	26.8 (15)
	Fleas	53 (I)	41.5 (22)	37.7 (20)
	Mites	22 (P)	27.3 (6)	31.8 (7)
	Ticks	47 (P/I)	32 (15)	29.8 (14)
	Total	436	36.2 (158)	36.9 (161)

Bacteria prevalence varied by locality anthropization types within ectoparasite groups. Notably, both agents showed higher detection in rural environments, e.g. *Bartonella* in fleas (55.9%) and mites (33.3%), and *Rickettsia* in mites (66.7%) and fleas (61%) (Table 4).

### Molecular identification and genetic diversity

**Figure 3.**
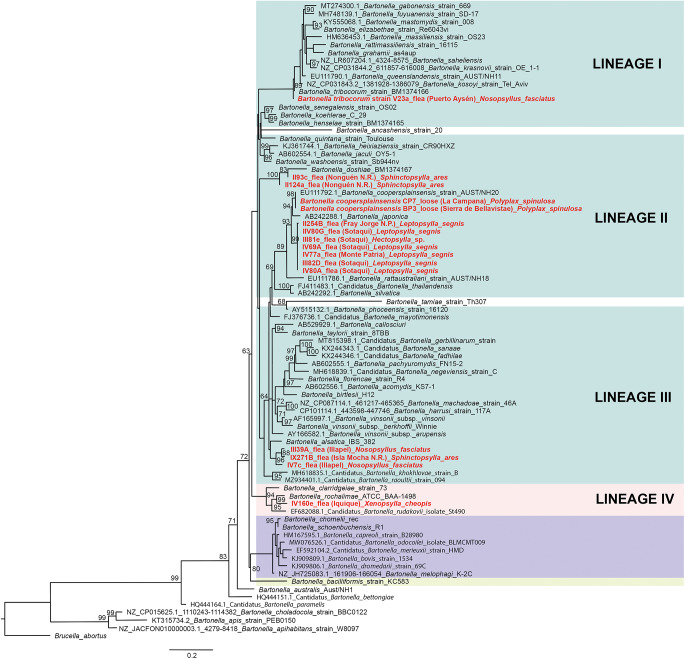
Phylogenetic tree obtained by maximum likelihood of *Bartonella* sequences based on the *rpoB* region (highlighted in red). These sequences were obtained from ectoparasites on *Rattus rattus* from various locations in Chile. The numbers at the nodes represent the Bootstrap support value (1000 replicates).

**Figure 4.**
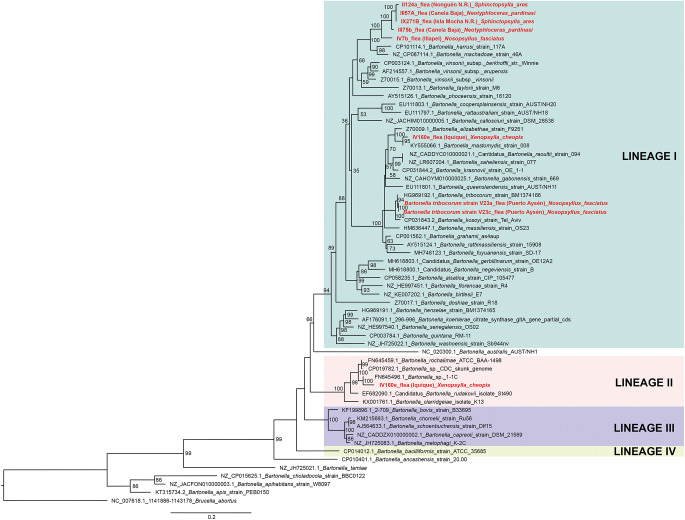
Phylogenetic tree obtained by Maximum Likelihood of *Bartonella* sequences based on the *gltA* region (highlighted in red). These sequences were obtained from ectoparasites on *Rattus rattus* from various locations in Chile. The numbers at the nodes represent the Bootstrap support value (1000 replicates).

**Figure 5.**
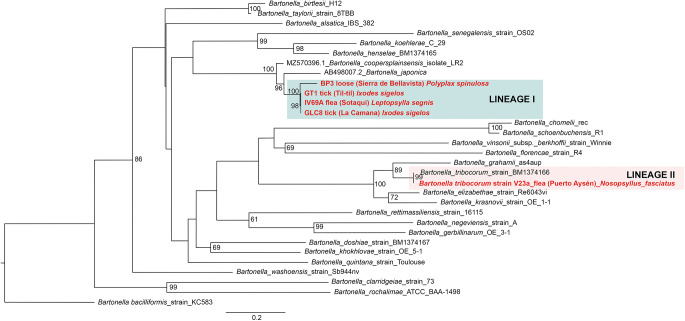
Phylogenetic tree obtained by Maximum Likelihood of *Bartonella* sequences based on the *ITS* region (highlighted in red). These sequences were obtained from ectoparasites on *Rattus rattus* from various locations in Chile. The numbers at the nodes represent the Bootstrap support value (1000 replicates).

**Figure 6.**
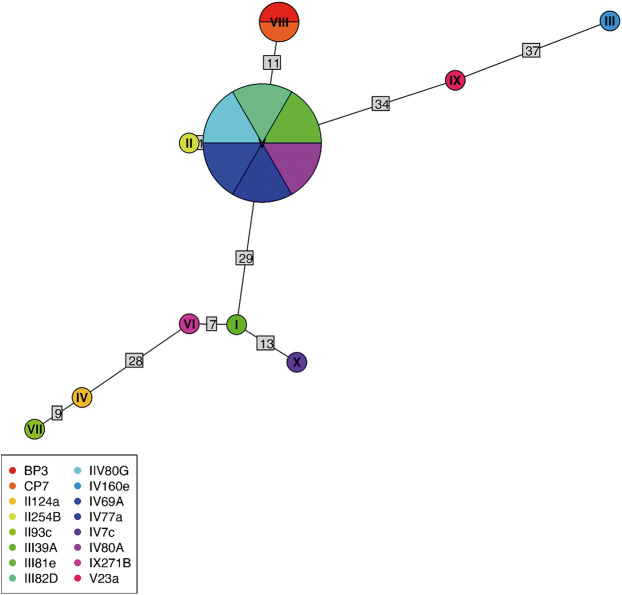
Haplotype network for *Bartonella* using sequences from the *rpoB* region extracted from ectoparasites of *Rattus rattus* in different locations in Chile. The numbers on each branch indicate the number of nucleotide changes between haplotypes. BP3, lice from Sierra de Bellavista; CP7, lice from La Campana; II124a, flea from R. N. Nonguén; II125B, flea from P. N. Fray Jorge; II93c, flea from R. N. Nonguén; III39A, flea from Illapel; III81e, flea from Sotaquí; III82D, flea from Sotaquí; IIV80G, flea from Sotaquí; IV160e, flea from Iquique; IV69A, flea from Sotaquí; IV77a, flea from Monte Patria; IV80A, flea from Sotaquí; IX271B, flea from R. N. Isla Mocha; V23a, flea from Puerto Aysén.

**Figure 7.**
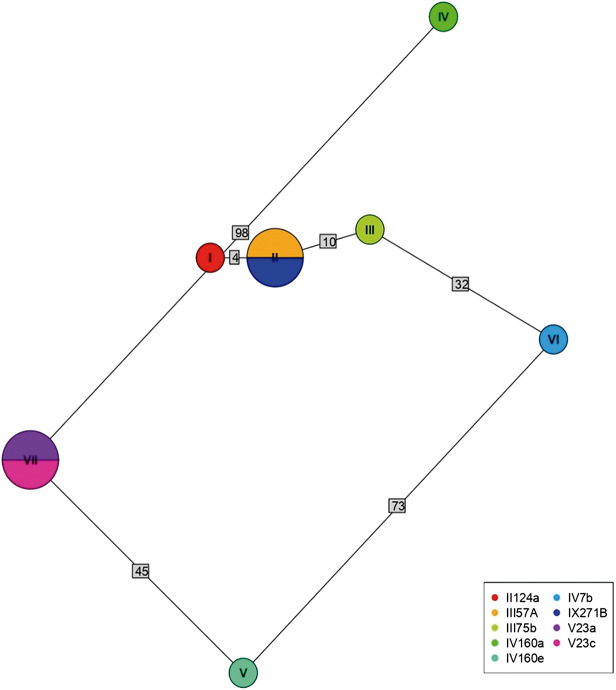
Haplotype network for *Bartonella* using sequences from the *gltA* region extracted from ectoparasites of *Rattus rattus* in different locations in Chile. The numbers on each branch indicate the number of nucleotide changes between haplotypes. II124a, flea from R. N. Nonguén; III57A, flea from Canela Baja; III75b, flea from Canela Baja; IV160a, flea from Iquique; IV160e, flea from Iquique; IV7b, flea from Illapel; IX271B, flea from R. N. Isla Mocha; V23a, flea from Puerto Aysén; V23c, flea from Puerto Aysén.

**Figure 8.**
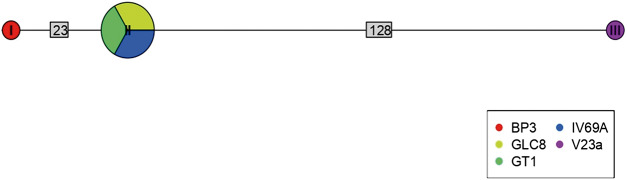
Haplotype network for *Bartonella* using sequences from the *ITS* region extracted from ectoparasites of *Rattus rattus* in different locations in Chile. The numbers on each branch indicate the number of nucleotide changes between haplotypes. BP3, lice from Sierra de Bellavista; GLC8, tick from La Campana; GT1, tick from Til–Til; IV69a, flea from Sotaquí; V23a, flea from Puerto Aysén.

Of 68 amplified *Bartonella* targets (10 *gltA*, 35 *rpoB* and 23 *ITS*), 30 yielded high-quality sequences suitable for analysis. Six samples provided multilocus confirmation (e.g. BP3, II124a, IV160e), with consistent taxonomic assignments across loci (Table 5). Sequences of *gltA, rpoB* and *ITS* of *Bartonella* isolated from fleas revealed significant divergence, associated with *B. tribocorum, B. rochalimae, B. mastomydis, B. doshiae* and several uncultured *Bartonella* lineages (Figures 3–5). On the other side, *rpoB* and *ITS* showed that sequences obtained from *P. spinulosa* were associated to *B. coopersplainsensis* and *B. japonica* (Figures 3 and 5). Meanwhile, *ITS* sequences from ticks were associated in the same clade of *Bartonella* of fleas and lice (Figure 5). Haplotype analysis in *Bartonella* seuqences from *rpoB*, *gltA* and *ITS* regions revealed five haplotypes for *rpoB*, seven for *gltA* and three for *ITS*. Haplotype diversity values were high across all markers (Hd-*rpoB*= 0.87, Hd-*gltA*= 0.94, Hd-*ITS*= 0.70), indicating substantial genetic variability despite the limited sample size (Figures 6–8).

**Table 5.** BLASTn results for *Bartonella* and *Rickettsia* sequences obtained from ectoparasites of *Rattus rattus*. The table indicates gene fragments, number of occurrences, closest BLASTn matches, similarity values, and GenBank accession numbers. An additional column specifies the samples that yielded multilocus sequences (*gltA, rpoB*, *ITS*)

Gene	Occurrence	BLASTn Identity	Similarity (%)	GenBank Access Numbers	Sample code (multilocus)
*Bartonella*					
*gltA* (*n* = 9)	6	*Bartonella* sp. *gltA* gene (strain C1phy)	95–98.95	Z70022.1, CP019489.1	II124a, IV160e, IX271B
	3	*Bartonella tribocorum*	99.28–100	OP382446.1, OQ191804.1	–
ITS (*n* = 5)	4	Uncultured *Bartonella* sp.	95.23–100	OQ190409.1	BP3, IV69A, IX43
	1	*Bartonella tribocorum*	99.81	OQ190411.1	–
*rpoB* (*n* = 16)	2	*Bartonella coopersplainsensis*	100	MF105900.1	–
	1	*Bartonella doshiae*	92.81	AF165991.1	–
	8	Uncultured *Bartonella* sp.	95.33–99.07	ON394035.1, GU338939.1	BP3, II124a, IV160e, IV69A, IX271B, IX43
	4	*Bartonella* sp.	93.77–98.13	LC596945.1, CP019489.1	–
	1	*Bartonella tribocorum*	100	MH748138.1	–
*Rickettsia*					
*gltA* (*n* = 1)	1	*Rickettsia* sp.	99.71	KY753118.1	–
*ompB* (*n* = 31)	31	*Rickettsia felis*	99.12–100	GU182892.1, MN267050.1	–

**Figure 9.**
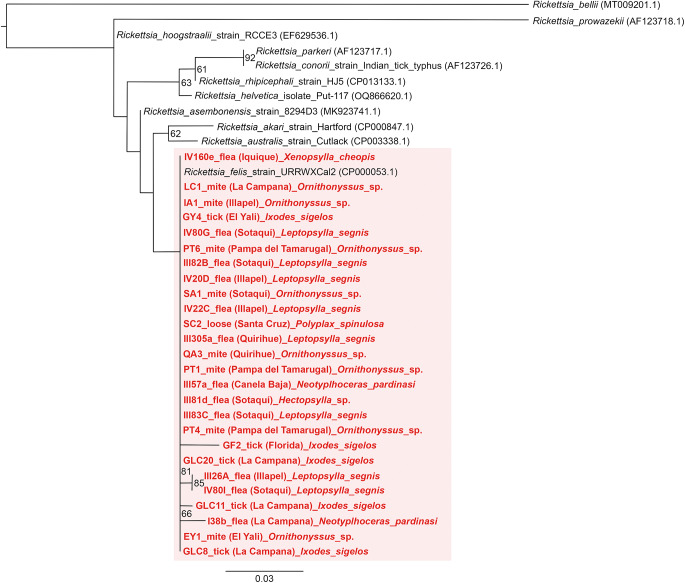
Phylogenetic tree obtained by Maximum Likelihood of *Rickettsia* sequences based on the *ompB* region (within the pink box). These sequences were obtained from ectoparasites on *Rattus rattus* from various locations in Chile. The numbers at the nodes represent the Bootstrap support value (1,000 replicates).

For *Rickettsia*, 31 high-quality *ompB* sequences were obtained, all matching *Rickettsia felis* (99, 12–100% identity to GenBank MN267050.1 in 30 cases). A single *gltA* sequence from a flea in Illapel showed 99.7% identity to an uncultured *Rickettsia* sp. (GenBank accession: KY753118.1) previously reported in Brazil (Table 5). The *ompB* phylogenetic tree revealed a polytomy among sequences of fleas, mites, lice and ticks across multiple localities, all of them associated only with *R. felis* (Figure 9).

### Association with environmental and parasitological descriptors

Generalized linear models revealed negative associations between pathogen occurrence and certain ecological variables (Table 6). The presence of *Bartonella* was significantly reduced with increasing anthropization (β = –0.48, *p* = 0.005) and higher tick prevalence (β = – 0.42, *p* = 0.009). Similarly, the presence of *Rickettsia* was lower with higher flea prevalence (β = –0.12, *p* = 0.02) and tick prevalence (β = –0.30, *p* = 0.01) were both negatively associated with detection. Neither bacterium showed significant associations with overall ectoparasite abundance or diversity.

**Table 6.** Generalized linear models (GLM) of *Bartonella* and *Rickettsia* presence. Values that are statistically significant are show in bold

Dependent variable	Modell performance			Factors	Estimate (β)	SE	L–R *χ*^2^	*p*-Value
	L-R *χ*^2^	*df*	Prob > *χ*^2^					
*Bartonella* presence	125.55	10	< 0.0001*	Intercept	55.37	8.09	46.81	< 0.0001*
				Anthropization	–	0.17	7.71	0.005*
				Flea abundance	0.13	0.18	0.52	0.47
				Lice abundance	–	0.09	3.20	0.07
				Mite abundance	–	0.21	0.34	0.56
				Tick abundance	0.87	0.52	2.72	0.09
				Ectoparasite diversity	–	4.08	0.03	0.85
				Flea prevalence	0.02	0.07	0.06	0.81
				Lice prevalence	0.07	0.11	0.38	0.54
				Mite prevalence	–	0.15	0.03	0.85
				Tick prevalence	–	0.16	6.74	0.009*
*Rickettsia* presence	96.59	10	< 0.0001*	Intercept	39.98	6.07	43.34	< 0.0001*
				Anthropization	–	0.15	1.01	0.31
				Flea abundance	–	0.16	2.63	0.10
				Lice abundance	–	0.05	0.004	0.95
				Mite abundance	0.02	0.17	0.01	0.90
				Tick abundance	0.16	0.33	0.23	0.63
				Ectoparasite diversity	2.09	3.11	0.45	0.50
				Flea prevalence	–	0.05	5.29	0.02*
				Lice prevalence	0.01	0.11	0.01	0.91
				Mite prevalence	–	0.10	0.61	0.43
				Tick prevalence	–	0.12	6.04	0.01*

L-R, likelihood ratio; *df*, degrees of freedom; SE, standard error;

* *p* ≤ 0.05.

## Discussion

This study evaluated the association between ectoparasite abundance, prevalence and diversity – including lice, fleas, mites and ticks – and the presence of *Bartonella* and *Rickettsia* in *R. rattus* across urban, rural and wild environment in Chile. Contrary to our hypothesis, anthropization and ectoparasite prevalence showed negative associations with bacteria detection: *Bartonella* occurrence declined whit increasing urbanization and higher ticks prevalences, while *Rickettsia* was negatively associated with both flea and tick prevalences. These results suggest complex ecological interactions that may involve vector competition, host immune modulation, or environmental filter.

### Ectoparasites and degree of anthropization

Lice (*P. spinulosa*) were the most abundant ectoparasites overall and predominated in wild areas, in contrast with the higher urban dominance of *N. fasciatus* and rural prevalence of *L. segnis*. These results contrast with those reported by Ho et al. (2021), who conducted a systematic review and meta-analysis of ectoparasites in synanthropic rodents, including urban rats, and found that mites were the most common ectoparasites (42.6% prevalence), followed by ticks (21.5%), lice (17.8%) and fleas (14.1%). Nevertheless, the study emphasized the considerable variability in prevalence estimates across studies, largely influenced by climatic conditions, which in turn shaped by the geographic regions where the research was conducted. Similar ectoparasites trend have been described in South America Mediterranean and temperate climates (e.g. Lareschi et al., 2019; Alonso et al., 2020), which are more closely the environmental context of our study areas. Consistent with Ho et al. (2021) our estimates also showed wide confidence intervals, reflecting high heterogeneity in ectoparasite loads.

Other studies conducted in urban and rural areas support the high prevalence of lice and fleas in rats (Fagir and El-Rayah, 2009; Mlik et al., 2022), with *Polyplax* spp. commonly dominating infestations (Chan et al., 2024). In our study, *P. spinulosa* was indeed the most prevalent louse species, but contrary to expectations, it was most abundant in wild rather than rural or urban areas. This may reflect poorer host conditions in wild environment, where individuals limited food availability could suppress immune responses and favour higher louse burdens (Chan et al., 2024).

Although lice are typically host-specific, *P. spinulosa* exhibits remarkable host plasticity, having been recorded on 13 *Rattus* species and other murids (*Bandicota, Leggadina, Mesembriomys, Pseudomys*), as well as a marsupial (*Didelphis*) (Durden and Musser, 1994; Wang et al., 2020; Ruiz et al., 2025). The transfer and establishment of this louse have been documented in endemic rats in Australia; however, these cases of transfer have only been documented among species belonging to Muridae, subfamily Murinae (Wang et al., 2020). Recent detection of this louse on *Didelphis albiventris* in rural Argentina, where invasive rats and native marsupials coexist, highlights its potential for cross-order spillover (Ruiz et al., 2025). In Chile, there are no native representatives of Muridae, which makes parasite transfer to wild species unlikely. However, the high prevalence in rat populations, together with frequent contact with native rodents and marsupials in shared habitats, could still increase the probability of transfer across phylogenetically distant hosts (Ruiz et al., 2025).

Fleas displayed the highest species richness, with most taxa previously associated with native Chilean rodents (Moreno-Salas et al., 2019, 2020). Only 3 species – *L. segnis, N. fasciatus* and *X. cheopis* – are typically linked to *Rattus. Xenopsylla cheopis* was found exclusively in Iquique (northern Chile) a coastal desert city, suggesting that arid conditions may restrict its establishment elsewhere despite the widespread distributions of rats.

While *L. segnis* and *N. fasciatus* showed comparable overall prevalence and abundance, their spatial distribution differed markedly: *L. segnis* was most prevalent in rural areas, whereas *N. fasciatus* dominated in cities. This may reflect host preferences, *L. segnis* is commonly associated with *M. musculus*, while *N. fasciatus* favours *R. rattus* and *R. norvegicus*, and the higher density and interspecific contact among synanthropic rodents in urban settings (Fitzgerald et al., 2023).

Among the native fleas, *Sphinctopsylla ares*, typically parasitizing *Abrothrix* spp. was frequently found on *R. rattus* in wild and rural sites, likely due its generalist feeding behaviour and broad distribution (Beaucournu et al., 2014).

In contrast, ticks and mites showed no significant variation across anthropogenic gradients, suggesting a more uniform distribution and ecological flexibility (Paramasvaran et al., 2009; Modi and Vankara, 2021). *Ornithonyssus* sp. was the most prevalent and abundant mite genus. In rats, the typical species described is *O. bacoti*; however, morphological similarity within the *Ornithonyssus* complex prevented species-level identification. Molecular barcoding was not feasible due to resources constraints, a recognized limitation. This genus is recognized as a potential biological vector of pathogens affecting wildlife, domestic animals and humans (Sargison et al., 2025). In Chile, *O. bacoti* has been recorded in rats and linked to cases of dermatitis (Barriga and Donckaster, 1965; Jofré et al., 2009). Notably, *Ornithonyssus* sp. has also been detected on native cricetid rodents and marsupials (Silva-de la Fuente, 2019), raising questions about cross-transmission between invasive and native host.

Regarding ticks, *I. sigelos* was the most abundant species. Its presence on *R. rattus* aligns prior reports (Osorio, 2001) and underscore that rat´s role as a potential dispersal host, even into urban areas (González-Acuña et al., 2004). Given that *I. sigelos* also parasitizes native rodents, shared habitats may facilitate parasite exchange between invasive and endemic fauna. Additionally, 2 specimens of *Ornithodoros* sp. were collected from rats in La Campana National Park. Although species-level identification was not possible due to specimen damage, they likely represent the soft tick known to parasitize the endemic rodent *Octodon degus* in the regions (Muñoz-Leal et al., 2020). Unfortunately, insufficient material precluded molecular confirmation.

Overall, local environment conditions, including host density, landscape configuration and interspecific interactions, strongly influence ectoparasite dynamics (Brunner and Ostfeld, 2008; Young et al., 2015; Krasnov et al., 2022; Klain et al., 2023). Although *R. rattus* is closely associated with human structures, which may homogenize microhabitats across urban and rural sites, wild areas remained ecologically distinct, supporting unique parasite-host interactions.

### Detection and prevalence of *Bartonella* and *Rickettsia*

*Bartonella* and *Rickettsia* DNA were detected in all ectoparasite groups investigated. Notably, prevalence estimates must be interpreted according to analytical method: fleas and adult ticks were processed individually, whereas lice, mites and immature ticks (larvae and nymphs) were analysed in pools of up to 10 specimens per host. Consequently, prevalence values for the latter groups reflect pool-level positivity rather than individual infection rates – a distinction critical for cross-study comparisons.

Fleas exhibited the highest *Bartonella* prevalence (53.4% in individually screened specimens), exceeding values reported in Thailand (25.8%; Klangthong et al., 2015), Chile (21.3%; Moreno-Salas et al., 2019) and Southeast China (26.2%; Zhou et al., 2024). This elevated detection likely reflects both the sensitivity of our qPCR approach and the use of individual screening, which avoids the dilution effects inherent in pooled designs.

Lice (*P. spinulosa*) showed a *Bartonella* prevalence of 33.3% (pool-level), aligning with Reeves et al. (2006), who reported ∼30% in pooled lice from Egyptian *Rattus* spp. This pattern is further supported by Klangthong et al. (2015), who found 57.1% prevalence in pooled rodent lice (*Polyplax* and *Hoplopleura* spp.) in Thailand. The consistency of high *Bartonella* detection across geographically distant studies using similar pooling strategies suggests that lice are significant and consistent reservoirs or vectors in synanthropic rodent systems.

A particularly novel finding was the detection of *Bartonella* in 26.6% of *I. sigelos* – a native Chilean tick primarily associated with wild rodents such as *Abrothrix* spp. and *Octodon degus* (González-Acuña et al., 2004). To our knowledge, this represents the first molecular detection of *Bartonella* in *I. sigelos*. This contrasts sharply with a recent large-scale U.S. field study that found *Bartonella* in only 1 of 853 blood-fed *Ixodes* ticks, despite high rodent bacteremia (Bai et al., 2024), suggesting that natural acquisition by *Ixodes* is rare in many regions. Our result may reflect either a regionally distinct host-vector-pathogen interaction in southern South America or be influenced by our pooling of immature stages, which can inflate apparent prevalence if only 1 individual per pool is infected.

*Bartonella* prevalence was highest in rural environments, particularly in fleas (55.9%) and mites (33.3%), a pattern also observed in Slovakia (80%; Špitalská et al., 2022) and central Chile (34.8%; Moreno-Salas et al., 2019), highlighting rural interfaces as key hotspots for pathogen circulation.

*Rickettsia felis* DNA was detected across all ectoparasite groups, with the highest prevalence in fleas (50.3% in individuals) – the highest among all vector groups examined. This aligns with reports from Chile (35.1%; Moreno-Salas et al., 2020) and Germany (28.6%; Obiegala et al., 2016), and falls within the range documented in U.S. *R. norvegicus* fleas (10–32%; Abramowicz et al., 2011). As with *Bartonella*, the high prevalence likely stems from our use of sensitive qPCR on individual fleas, avoiding signal dilution.

A novel finding was the detection of *R. felis* in 33.3% of *P. spinulosa* pools – the first report of *Rickettsia* in this louse species and, to our knowledge, the first documentation of *R. felis* in any rodent-associated louse globally. Previous studies identified *Rickettsia* only in *H. pacifica* (*R. typhi*; Reeves et al., 2006) and *P. serrata* (*R. helvetica*; Aleksandravičienė et al., 2021), but not in *P. spinulosa*. Reeves et al. (2006) explicitly screened *P. spinulosa* from Egyptian *Rattus* and found no *Rickettsia*, underscoring the novelty of our detection.

In mites (*Ornithonyssus* sp.), *R. felis* was detected in 23.2% of pools, reinforcing growing evidence that mesostigmatid mites may play an underappreciated role in *Rickettsia* ecology. This aligns with Reeves et al. (2007), who reported *Rickettsia* genotypes related to the Akari group – including *R. akari*-like strains – in *O. bacoti* collected from *Rattus* spp. in Egypt. Behera et al. (2023) also detected *Rickettsia* DNA (unspecified at the species level) in *O. bacoti* infesting *Mus* spp. in India, further supporting the role of this mite genus as a potential host for rickettsiae. Collectively, these studies – combined with our detection of *R. felis* in *Ornithonyssus* sp. from *R. rattus* in Chile – suggest that *Ornithonyssus* mites may contribute to *Rickettsia* circulation at sylvatic and peri-domestic interfaces, particularly in regions where invasive rodents interact with native fauna and human settlements.

*Rickettsia felis* was also detected in *I. sigelos*. This finding aligns with a growing body of evidence from across the globe, where *R. felis* has been molecularly detected in *I. ovatus, I. granulatus, I. hexagonus* and *I. ricinus* in Japan, Taiwan, Italy, Germany, France, Spain, Romania, Serbia and Slovakia (Tsui et al., 2007; Dobler and Wölfel, 2009; Pascucci et al., 2019; Banović et al., 2021; Borsan et al., 2021; Lejal et al., 2019). Critically, Danchenko et al. (2022) recently succeeded in culturing *R. felis* from a questing *I. ricinus* nymph in Slovakia, providing the first definitive evidence that this bacterium can not only be acquired by *Ixodes* ticks but also persist and replicate within them, thereby fulfilling a key criterion for vector competence. Our detection of *R. felis* in the native Chilean tick *I. sigelos* may therefore represent a genuine, albeit infrequent, natural infection, potentially facilitated by local ecological or host-specific factors. However, given that our tick samples included pooled immature stages, the possibility of transient carriage or signal contamination cannot be entirely excluded, and future studies using individual tick screening and bacterial viability assays are warranted.

*Rickettsia* prevalence was also highest in rural areas, especially in mites pool (66.7%) and fleas (61%), reinforcing the role of rural interfaces – where synanthropic rodents, native wildlife and humans converge – as key zones for enzootic maintenance and potential spillover.

### Molecular identification and genetic diversity

High-quality sequences were obtained for 30 *Bartonella* amplicons and 32 *Rickettsia* amplicons. BLAST and phylogenetic analyses revealed multiple zoonotic *Bartonella* species, including *B. tribocorum, B. rochalimae, B. coopersplainsensis, B. doshiae* and several uncultured lineages – many previously reported in Chilean rodents and their fleas (Moreno-Salas et al., 2019; Müller et al., 2020; Sepúlveda-García et al., 2023). Six samples yielded multilocus confirmation (e.g. BP3, II124a, IV160e), with consistent taxonomic assignments across loci.

For *Rickettsia*, 30 of 31 high-quality *ompB* sequences matched *R. felis* (100% identity to GenBank MN267050.1). A single *gltA* sequence from Illapel showed 99.7% identity to an uncultured *Rickettsia* sp. previously reported in Brazil (KY753118.1). Critically, *R. felis* was detected in fleas, lice, mites and ticks, indicating a broad ectoparasite range in Chile.

Despite this taxonomic breadth, *R. felis* exhibited remarkably low genetic diversity: the *ompB* haplotype network revealed 1 dominant haplotype (H1) in 30 of 31 sequences across all ectoparasite groups and localities (Hd = 0.06), mirroring patterns in Chilean foxes (Millán et al., 2023). This suggests clonal expansion of a single, well-adapted *R. felis* strain in Chilean synanthropic systems. In contrast, *Bartonella* showed higher haplotype diversity across *gltA, rpoB* and *ITS*, consistent with greater strain heterogeneity in rodent reservoirs (Kosoy and Bai, 2019).

### Association between *Bartonella* and *Rickettsia* presence with parasitological descriptors and degree of anthropization

Contrary to our initial hypothesis – that ectoparasite abundance, prevalence and diversity would positively correlate with pathogen detection – generalized linear models revealed significant negative associations between both *Bartonella* and *Rickettsia* occurrence and key ecological variables.

*Bartonella* presence was negatively associated with increasing anthropization and higher tick prevalence. This suggests that rural and wild environments – rather than urban centres – serve as more favourable settings for *Bartonella* circulation in *R. rattus* ectoparasite communities. This pattern aligns with evidence that *Bartonella* enzootic cycles are sensitive to habitat fragmentation and anthropogenic disturbance, which may disrupt stable rodent – vector networks required for bacteria maintenance (Kosoy and Bai, 2019).

In contrast, *Rickettsia* occurrence showed no association with anthropization, but was negatively associated with both flea prevalence and tick prevalence. This decoupling from urbanization likely reflects the well-documented adaptability of *R. felis* to peri-domestic and synanthropic systems worldwide (Gillespie et al., 2009). In Chile, *R. felis* has already been detected in fleas from wild foxes across Mediterranean landscapes, indicating its capacity to persist across environmental gradients (Millán et al., 2023).

These counterintuitive patterns indicate that pathogen dynamics are not driven by simple metrics of ectoparasite load, but rather by complex ecological filters such as host community composition, vector competence, or competitive exclusion among co-occurring ectoparasites. Critically, given the cross-sectional design of our study, no causal relationships can be inferred from these statistical associations.

Notably, overall ectoparasite abundance and diversity were not significantly associated with the presence of either pathogen, reinforcing that taxon-specific interactions, rather than general parasitism intensity, shape pathogen occurrence in this system. Critically, no significant associations were found with overall ectoparasite abundance or diversity, reinforcing that taxon-specific interactions, rather than general parasitism intensity, drive pathogen dynamics in this system.

Future work incorporating strain-level resolution, vector identification at the species level, and longitudinal sampling will be valuable to determine whether these negative associations represent stable ecological relationships or temporal fluctuations. Overall, our results underscore the complex interplay between rodents, ectoparasites and bacterial pathogens in central Chile. Anthropogenic disturbance was negatively associated with *Bartonella* presence, whereas *Rickettsia* occurrence was more strongly linked to the distribution of specific ectoparasite taxa. Given the detection of zoonotic lineages and uncharacterized strains, continued molecular surveillance is essential to understand and mitigate the public-health risks associated with ectoparasite-borne pathogens in synanthropic environments.
